# Key metrics for monitoring performance variability in edge computing applications

**DOI:** 10.1186/s13638-025-02469-6

**Published:** 2025-05-30

**Authors:** Panagiotis Giannakopoulos, Bart van Knippenberg, Kishor Chandra Joshi, Nicola Calabretta, George Exarchakos

**Affiliations:** 1 Electrical Engineering, Eindhoven University of Technology, Eindhoven, The Netherlands; 2 Materials and Structural Analysis, Thermo Fisher Scientific, Eindhoven, The Netherlands

**Keywords:** Edge computing, Performance variability, Monitoring metrics, Kubernetes, Prometheus

## Abstract

Edge computing is an emerging approach that enables applications to run closer to users, accommodating their specific execution time requirements. Edge computing systems typically consist of heterogeneous processing and networking components, resulting in inconsistent task performance. To improve the consistency of edge computing applications, this study presents a method to identify the factors that affect variability in task execution time. We deploy a set of single-particle analysis algorithms, designed for an electron microscopy use case, running on a Kubernetes cluster monitored by Prometheus. This specific usecase was chosen because it encompasses a diverse set of time-sensitive and privacy-sensitive applications, with a wide range of resource requirements. Our experiments revealed a significant increase in the variability of round-trip time when tasks share resources. The proposed approach identifies the most relevant monitoring metrics from a larger set of collected ones (provided by Prometheus), with correlations up to 87%. This process reduces the number of metrics to 90, achieving a reduction of 80%. As a result, the overhead of the monitoring system is decreased, and the use of these metrics for further processing, such as predictive modeling and scheduling, is simplified. These selected metrics not only help to understand the causes of performance variability, but also possess predictive value, enabling more efficient scheduling. The prediction power of these metrics is shown using SHapley Additive exPlanations analysis.

## Introduction

With the increasing demand for low latency, high reliability, and enhanced privacy in modern applications, edge computing has emerged as a promising solution to meet these requirements. It involves placing computing resources close to data sources and users. Unlike traditional data centers, edge computing uses small distributed infrastructures, which can lead to competition for resources among applications. Therefore, careful planning of computing and network resources is necessary to ensure predictable performance, efficient use of resources, and autonomous management of the edge infrastructure. Among the potential usecases of edge computing includes cloud gaming, autonomous networks, patient monitoring, and virtualized network functions in disaggregated 5G/6G networks requiring predictable execution times to meet service-level agreements (SLAs). These SLAs specifically focus on the request completion time of the application as experienced by end users.

Placing applications together in a distributed computing and network resource pool can lower operational expenses (OPEX), improve resource use [1], and reduce average execution times [2]. However, this approach also results in variability in the execution times of co-located applications [3]. This makes proactive resource allocation a challenging task [4, 5]. To effectively manage this variability, it is crucial to identify those monitoring metrics (e.g., CPU usage, memory status, network load) that reflect the performance of the system and the execution time of each task.

In this paper, we study the performance variability of an edge computing system, focusing on electron microscopy (EM). In this field, reliability, privacy, and latency are crucial. Our study focuses on the single-particle analysis (SPA) acquisition and processing workflow, a typical example of real-time EM instrument control and adjustment. During SPA acquisition, thousands of 2D image stacks of a vitrified sample are collected. These 2D projections are subsequently processed to reconstruct the 3D structure of the molecule [6]. The computing and network resources in a typical EM system are used heavily during SPA operations or remain idle when SPA is not active. To address this, we propose creating a distributed pool of computing and networking resources in EM farms. The goal is to provide cost-effective resources for SPA analysis while meeting strict latency, reliability, and data privacy requirements. SPA includes a variety of applications, each with different resource needs (i.e., CPU, GPU, network), allowing us to study a wide range of application types.

We have developed a framework for automatically analyzing performance fluctuations due to resource sharing among applications and identifying sources of variability. Our approach uses a Kubernetes-managed [7] computing cluster with GPU units, monitored by Prometheus [8]. By applying a feature extraction technique to the time-series data from the monitored metrics, we examine the correlations between the state of these metrics during task execution and the round-trip time (RTT) of edge computing tasks. Specifically, we analyze a large set of monitoring metrics (i.e., 422 provided by Prometheus) and identify the most correlated with fluctuations of the target performance metric, particularly RTT. The number of monitoring metrics provided can vary, given the installed version and the type of Prometheus agents. Our method detects and filters the most significant parameters and factors causing or related to variability. As a result, further processing of the monitoring metrics (i.e., building predictors) become less complex and faster as they rely on a reduced, yet highly relevant subset of input variables. The predictive power of the identified monitoring metrics to forecast RTT is demonstrated using SHapley Additive exPlanations (SHAP) analysis. Our findings indicate that the RTT of different co-located applications correlates with a specific subset of resource monitoring metrics. Therefore, it is necessary to create individual profiles for each application, which include the subset of monitoring metrics that are most strongly correlated with the performance variability of that application. We investigate how performance variability and the most important metrics change when applications are hosted on different servers. Finally, we demonstrate how various configurations of our methodology impact the accuracy of capturing performance variability and discuss its limitations.

The remainder of this paper is organized as follows: Section 2 reviews the relevant literature. Section 3 explains the methodology used to correlate monitoring metrics with performance. Section 4 describes the experimental setup. Section 5 presents the results of the experiments. Finally, Sect. 6 provides a summary of the conclusions and directions of the future work.

## Related work

**Table 1:** Comparison of related work and our contributions

Work	Key findings	Comparison with our work
[9]	Analyzed performance variability caused by the OS, hardware, and application. Evaluated isolated executions with some shared network analysis. Proposed strategies to mitigate variability.	Analyzed performance variability cause by the co-location of applications. Evaluated shared compute and network resources. Proposed the use of monitoring metrics as performance indicators.
[4]	Investigated performance variability in memory, network, and disk on isolated servers using benchmarks. Identified sources of variability.	Investigated performance variability in CPU, memory, disk, network, and GPU on shared servers. Identified most correlated metrics to performance fluctuations during application co-location.
[5]	Examined CPU performance variability on isolated servers using benchmarks. Identified sources of variability of CPU.	Examined CPU, memory, disk, network, and GPU variability on shared servers. Identified most correlated metrics to performance fluctuations during application co-location.
[3]	Analyzed performance variability of applications under shared execution. Found that specific application placements lead to better performance.	Analyzed performance variability for different application placements. Found that application profiles require distinct set of monitoring metrics (i.e., for scheduling or predictions).
[10]	Investigated the impact of system configuration changes (e.g., kernel version) on performance. Used benchmarks in an isolated environment.	Investigated the correlation of monitoring metrics on performance of co-located applications. Used real-world applications in a shared environment.
[11, 12]	Applied anomaly detection to monitoring metrics to identify resource pool malfunctions.	Applied a correlation-based methodology to identify key monitoring metrics related to performance fluctuations. Reduced the number of monitoring metrics required post-processing (i.e., anomaly detection).
[2]	Developed execution time predictors by exposing application execution stages. Predictions were made after task submission.	Developed a methodology to identify key metrics correlated with performance fluctuations. Monitoring metrics provide performance insights before task submission.
[13]	Designed a Kubernetes network plugin for fast, deterministic inter-container communication.	Designed a methodology to identify reliable monitoring metrics across various resources (e.g., CPU, GPU), extending beyond network performance.

The key findings of previous work along with a comparison with our research are presented in Table 1. It is well studied that the performance of task execution in shared computing and networking infrastructure can vary significantly for the same task due to factors such as interference from other applications, hardware quality and hardware or software versions [14, 15]. In [14], the authors conducted an extensive study on task execution variability in high-performance computing (HPC) environments and highlighted the limitations of previous research in managing data generated by such systems. Chunduri et al. [9] explored the performance variability that occurs when software is executed multiple times on the same or different machines with varying hardware configurations. They identified the degree of variability caused by the operating system (OS), hardware components, and application level, proposing strategies to reduce it. Maricq et al. [4] took a similar approach and introduced the CONFIRM framework, which estimates the minimum number of repetitions needed to achieve a desired level of confidence in sample distributions using nonparametric confidence intervals [16]. Further research by Duplyakin et al. [5] extended this analysis to performance variability related to the CPU. Both papers [4, 5] concluded that the performance and variability differ between machines and system configurations.

Kuriata and Illikkal [3] advanced the field by studying performance variability in environments where multiple applications share the same host. Their research revealed that co-locating applications leads to performance degradation due to resource contention, especially when all applications are CPU-intensive. They also found that different types of co-located applications result in varying levels of performance variability. Although these studies provided valuable information on the causes of performance fluctuations, their primary focus was on reducing and controlling variability, rather than predicting it. In contrast, our approach treats variability as a fundamental characteristic of the system and leverages monitoring metrics to detect performance fluctuations with the aim of producing more reliable predictions and scheduling.

Zhao et al. [10] proposed a method to predict performance fluctuations by identifying changes in system state, such as updates to the OS or kernel versions, and used a gradient boosting model to predict the timing of these changes. Sukhija et al. [11] utilized Kubernetes, Prometheus, and Grafana to analyze monitoring metrics such as CPU utilization spikes, allowing real-time detection, analysis, and resolution of system incidents. This approach allows automatic anomaly detection in monitoring metrics, timely alerts for system administrators, and prevention of performance degradation. Mart et al. [12] developed a similar framework. While the works in [10–12] focus on detecting anomalies in system states, our research delves deeper into how monitoring metrics influence the prediction of performance fluctuations.

Several studies have enhanced existing resource allocation mechanisms, such as those in Kubernetes, by integrating advanced controllers to enable more efficient decision making. Fu et al. [2] estimated the remaining execution time of containers by exposing the execution stage of the deployed applications. This approach allowed containers to be placed on the most suitable nodes (i.e., those with minimal interference), significantly reducing the total completion time of applications. Garbugli et al. [13] developed KuberneTSN, a Kubernetes network plugin designed to establish an accelerated and deterministic communication channel between containers. This was achieved by eliminating the overhead of the kernel networking stack and introducing a Time-Sensitive Networking (TSN) standard-compliant packet scheduler. Building on these advancements, our research identifies monitoring metrics across all resource types (i.e., CPU, GPU, and network) that correlate with performance variations. These metrics can be used by scheduling mechanisms to improve decision making, leading to more efficient resource allocation.

## Methodology

The goal of our methodology is to automatically analyze a large set of monitoring metrics provided by the monitoring system and identify the most significant ones, specifically the top 10 correlated metrics with RTT. These key metrics offer valuable insights into application behavior and the underlying causes of performance variability. A “task” refers to a request sent to an application that remains active until a response is received. RTT measures the time elapsed between the initiation of the request by the client ($t_{\rm start}$) and the reception of the response ($t_{\rm end}$). The methodology can handle an arbitrary number of monitoring metrics. However, the total completion time of the analysis increases with the number of monitoring metrics provided. The current study focuses on identifying sources of performance variability within the cluster itself. For this reason, the input data used in our analysis do not introduce performance variability.

**Fig. 1:**
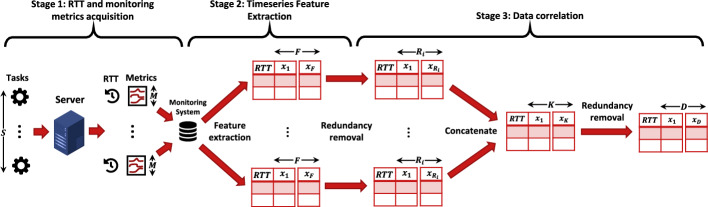
Stages of the proposed methodology to calculate the correlation between monitoring metrics and application performance

**Table 2:** Methodology annotation

Symbols	Description
*S*	Number of task repetitions
*M*	Number of available monitoring metrics
*F*	Number of extracted features by tsfresh
$R_i$	Number of extracted features for the $i_{th}$ metric after removing redundant features
*K*	Total number of extracted features from all *M* metrics
*D*	Number of extracted features after the final redundancy removal

Our research utilizes Kubernetes for orchestration and Prometheus for monitoring, but the approach is flexible enough to be adapted to other orchestration platforms and monitoring tools. The methodology consists of three main stages, as illustrated in Fig. 1. The first stage involves collecting RTT values and the corresponding monitoring metrics for analysis. In the second stage, these metrics are transformed into a format suitable for standard correlation techniques, such as Pearson correlation. Finally, in the third stage, the correlation algorithm is applied to identify the most critical metrics. By reducing the number of metrics, this method not only helps pinpoint the source of performance variability (e.g., CPU usage) but also reduces the complexity of subsequent processes, such as predictive model generation. The annotation used in our methodology is shown in Table 2.

### Stage 1: RTT and monitoring metrics acquisition

Evaluating the performance variability of a specific task requires repeatedly executing the task and measuring its RTT. The task executions may occur in isolation or alongside other applications, depending on their co-location. Performance variability is defined as the fluctuation in the performance of a task (i.e., RTT) over time. To estimate the minimum number of repetitions needed to achieve a desired confidence level, we use CONFIRM [4]. Specifically, we calculate the number of repetitions required to achieve $\alpha \%$ confidence (e.g., 95%) that the empirical median does not deviate by more than $r\%$ (e.g., 3%) from the true median of the RTT distribution. As the system operates at runtime, CONFIRM indicates when sufficient data have been collected to proceed with post-processing operations, such as calculating the correlation between monitoring metrics and RTT changes. Attempting to process the data before this point may result in incomplete or inaccurate analysis, potentially leading to invalid or misleading conclusions. Note that other performance metrics for each task can be used instead of RTT. The degree of variability is quantified using the Coefficient of Variation (CoV) for RTT, defined as ${\text{CoV}}_{{{\text{RTT}}}} = \frac{{\sigma _{{{\text{RTT}}}} }}{{\mu _{{{\text{RTT}}}} }}$. CoV is calculated when enough data samples are collected.

The state of the system (i.e., CPU, RAM, GPU, and network load) during the execution of tasks, within the interval $[t_{{{\text{start}}}}$, $t_{{{\text{end}}}} ]$, is captured through the recorded monitoring metrics. The network load uplink/downlink refers to the rate at which bytes are transmitted to or received from the server per second. The methodology is not limited to a specific number of monitoring metrics or a particular monitoring system. As a result, it seamlessly integrates with other monitoring systems by retrieving metrics directly from the relevant application programming interface (API). The number of the available monitoring metrics (i.e., 422) depends on the installed monitoring system, used version, and deployed monitoring agents. For example, servers equipped with a GPU run an additional monitoring agent specifically for GPU metrics. As a result, these servers expose a greater number of monitoring metrics (i.e., 422 metrics) compared to those without a GPU (i.e., 400 metrics). In addition, no modification of the application code is needed as all data are collected from the deployed monitoring system. Metrics that remain constant over time are excluded from the analysis, as they are not indicative of performance changes. For CPU-related metrics, values from multiple cores are aggregated by computing the average, reflecting the overall resource consumption on each server. The resulting dataset consists of *S* task repetitions (each with a unique ID) and the corresponding time-series values for each of the monitoring metrics *M*.

### Stage 2: time-series feature extraction

Time-series metrics represent a sequence of recorded values (e.g., memory usage) at specific time intervals. Thus, the monitoring metrics cannot be directly used by conventional correlation methods (i.e., Pearson correlation). To extract meaningful features from these time-series, we employ the tsfresh library [17]. tsfresh is an open-source Python package designed to automatically extract relevant characteristics from time-series data. It offers a broad set of statistical, temporal, and frequency domain features that are particularly suited for creating predictive models based on time-series analysis. The features extracted by tsfresh include measures such as mean, standard deviation, kurtosis, autocorrelation, and Fourier coefficients, among others. More details on tsfresh features are available in [18]. This diversity enables a comprehensive analysis of both short-term and long-term behaviors of the data. In addition, the time-series transformation reduces dimensionality and organizes the data into a more usable format for easier analysis and further processing. tsfresh has been effective in various domains, including anomaly detection [18], sensor fault classification [19], and human activity recognition [20]. By applying this method, we transform each of the *M* time-series metrics into *F* statistical and temporal features, generating a total of 785 features per metric. These feature sets, derived from time-series data, allow us to capture important patterns, such as trends, volatility, and periodicity, which are crucial for understanding the dynamics of monitoring metrics. Once transformed, the extracted features for each metric are aligned with their corresponding RTT values, ensuring that both share the same dimensionality. This alignment allows for straightforward correlation analysis using conventional techniques to identify the most impactful metrics influencing application performance. The version used in this work is 0.19.0. The extraction of features using tsfresh, based on time intervals of the monitoring metrics, can be performed either in real-time or after all data samples have been collected. In real-time extraction, features are generated immediately for each newly obtained data sample as it becomes available. Alternatively, the feature extraction process takes place only after all required data samples have been collected.

### Stage 3: data correlation

The final stage of our methodology focuses on correlating monitoring metrics with RTT to identify the most relevant factors impacting performance variability. The extracted time-series features for each task are correlated with RTT using Pearson correlation [21]. We use the absolute correlation values, as we are interested in the strength of the relationship rather than its direction. Our approach is also compatible with other correlation techniques, such as Spearman’s rank correlation. The correlation process is broken down into the following substages:

**Substage 3a (Correlation per metric):** For each of the metrics *M*, we calculate the correlation between its extracted features *F* and the RTT of the tasks, generating a correlation matrix for each individual metric.

**Substage 3b (Redundancy removal per metric):** Using the correlation matrices generated in Substage 3a, we reduce the number of features by eliminating redundant ones. If two or more features exhibit high inter-correlation (e.g., above 90 percent), we retain only the feature that has the stronger correlation with RTT. This substage reduces the number of features from *F* to a smaller number $R_i$ for each metric, where $i \in [1, M]$ and $R_i \le F$.

**Substage 3c (concatenation of features):** The remaining features of all metrics are concatenated into a single dataset, resulting in a dataset with *S* rows (representing tasks) and *K* columns (where $K = \sum _{i=1}^{M} R_i$).

**Substage 3d (redundancy removal across the dataset):** Substages 3a and 3b are applied again to the concatenated dataset. This produces a new correlation matrix that considers the relationships between all features and the RTT. Another round of redundancy elimination is conducted to further reduce the dataset to *S* rows and *D* columns, where $D \le K$.

**Substage 3e (top metrics extraction):** For each monitoring metric, we determine its correlation with RTT by selecting the highest correlation value among its extracted features. The metrics are then ranked in descending order based on correlation, allowing us to identify the most important ones for further analysis.

## Experimental setup

In the following section, we present the applications, frameworks, and infrastructure used in our experiments.

### Applications

Our experiments deploy five SPA applications: *Upload*, *MotionCor2*, *FFT Mock*, *gCTF*, and *ctffind4*. Each application accepts a POST request containing the input image and configuration, executes its corresponding algorithm, and returns the result. The applications are designed to handle one task at a time and, for this study, are treated independently of each other.

#### Input images

Image data were collected from a vitrified EM grid containing a protein sample. A Glacios electron microscope, equipped with a Falcon4i detector and EPU software [22], was used for data acquisition. However, the actual experiments were performed offline in a compute environment separate from the microscope. All input files for applications are stored in MRC format, a widely accepted standard for storing EM image and volume data [23]. The data comprises MRC fraction files, which serve as inputs for the *MotionCor2* and *Upload* applications. A single integrated MRC image is used as input for the remaining applications. The MRC fraction files are 897MB in size, while the integrated files are 65MB. The dataset contains 98 images in total.

#### Upload

The *Upload* application transfers the input images to the target server. Upon successful file transfer, the server acknowledges storage completion, after which the uploaded files are further processed by the *MotionCor2* application.

#### MotionCor2

Due to drift and beam-induced motion, the quality of SPA images can degrade during acquisition [24]. To counteract this, SPA images are often recorded as movies of multiple frames, allowing motion correction before the frames are combined into a single image. *MotionCor2* v1.5.0 [25] was employed in our experiments to correct motion in the recorded image stacks, producing a single-frame MRC file for further analysis.

#### FFT mock

Several lightweight image processing algorithms help in SPA acquisitions by automating tasks and keeping the microscope optimized. Since some of these algorithms are proprietary, a mock version was implemented that simulates the same computational load. This mock algorithm processes a single MRC image by duplicating and shifting pixels to generate a new image. Then, it uses fast Fourier transform (FFT) and pixel-wise operations to estimate the induced shift.

#### gCTF

One common task in EM image post-processing is contrast transfer function (CTF) correction. This step estimates key optical parameters from the image, such as defocus, which can then be corrected to improve image quality and ensure accurate downstream analysis. For this, we used *gCTF* v1.18 [26], a GPU-accelerated real-time algorithm for CTF determination and correction.

#### ctffind4

In addition to *gCTF*, we also utilized *ctffind* v4.1.14 [27], which similarly extracts CTF information from MRC images. Unlike *gCTF*, however, *ctffind4* operates on a CPU-only basis.

### Frameworks

#### Kubernetes

Kubernetes [7] is an open-source platform that orchestrates distributed computational resources (nodes) and simplifies application deployment through pods, which are container-based execution units. These containers use technologies such as Docker [28] or containerd [29]. The Kubernetes master node manages the cluster and maps pods to resources, while the worker nodes host the pods and provide computing power. Our experiment used Kubernetes v1.28.2, CRI Docker 0.3.9 on Docker 24.0.2, and NVIDIA Container Runtime v1.13.5 for GPU support. Kubernetes resources include CPU, RAM, GPU, disk, and network. GPU time-slicing, managed via the NVIDIA GPU Operator, allows multiple tasks to share GPU resources.

#### Prometheus

Prometheus [8], integrated with Kubernetes, monitors applications and infrastructure by collecting metrics in real time. The Prometheus architecture consists of a server and various monitoring targets. The server regularly collects metrics from these targets in a text-based format over HTTP, storing the data in a local time-series database. Users can retrieve and visualize this data through the built-in web interface, dashboard tools such as Grafana [30], or by querying the HTTP API directly. The metrics include CPU, RAM, disk, network, and GPU usage. GPU metrics are extracted via the NVIDIA DCGM exporter [31]. The metrics fall into two categories: *gauge* (e.g., CPU usage) and *counter* (e.g., packet count). Counters are converted into gauge metrics by applying rate functions in Prometheus Query Language (PromQL). In our setup, we used Prometheus v2.54.0 and DCGM v2.4.6$-$2.6.9, with metrics recorded at intervals of 200 milliseconds.

### Compute infrastructure

**Fig. 2:**
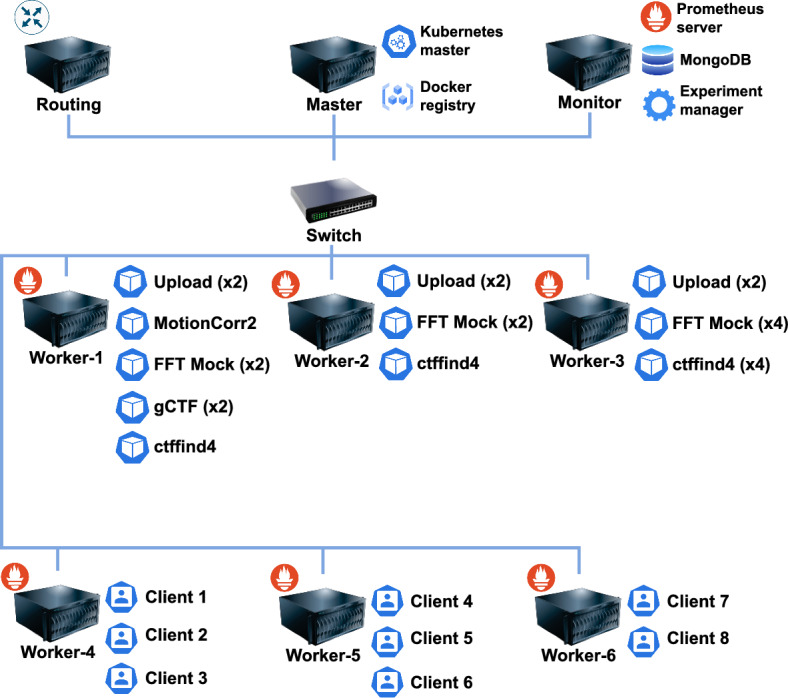
Structure of the used infrastructure. The blue lines indicate the physical connectivity of the servers through a 1 Gbps Ethernet link

The experimental setup is illustrated in Fig. 2, and the device specifications are shown in Table 3. The effectiveness of our methodology is independent of the setup size, as our analysis focuses on metrics per individual machine. Key considerations include system monitoring and isolation from external variability factors. We define three different co-location scenarios for Worker-1, Worker-2, and Worker-3, as depicted in Fig. 2. These scenarios are executed in parallel. The Kubernetes cluster comprises one master node and four worker nodes, all interconnected via 1 Gbps links. The Worker-1 is equipped with an NVIDIA GPU Tesla K20c.

The master node is responsible for managing the worker nodes, supervising the pods, and hosting the local Docker registry. The “Monitor” node hosts the Prometheus server, the experiment manager, and a MongoDB instance, which stores the experimental data. The experiment manager handles the deployment and termination of experimental pods by interacting with the Kubernetes API. The worker nodes are tasked with deploying applications and generating tasks. Worker-1, equipped with a GPU, runs GPU-based pods. Workers 1-3 are used to host application pods, while Workers 4–6 simulate clients in the form of pods. Each client sends a request to the target application, waits for the response, and then pauses for a duration defined as $t_\text{wait}$ before submitting a new task. The waiting time, $t_{wait}$, is randomly selected from the interval $[0,t_\text{max}]$, where $t_\text{max}$ is 40, 6, 20, 10, and 10 for *upload*, *ctffind4*, *FFT Mock*, *gCTF*, and *MotionCor2*, respectively. These values were experimentally determined to induce interference between applications. The RTT of each task is recorded on the client side and stored in MongoDB. Future work will focus on incorporating more realistic workload simulations. Resources allocated to each application are not restricted. At the end of the experiment process, RTT data and monitoring metrics are retrieved from MongoDB and Prometheus for analysis. The data are then processed to identify the most relevant monitoring metrics that contribute to RTT variability, as explained in Sect. 3.

**Table 3:** Specifications of infrastructure components

Name	Type	Model/Processor	Cores	RAM	Disk
Switch	Physical	Dell PowerConnect 5448	–	–	–
Routing	Virtual	Intel Xeon X3430	3	4 GB	HDD
Master	Virtual	Intel Xeon Silver 4109T	8	32 GB	SSD
Monitor	Virtual	Intel Xeon Silver 4109T	8	78 GB	SSD
Worker-1	Physical	Intel Core i7-7700	4	16 GB	HDD
Worker-2	Physical	Intel Core i5-9600	6	16 GB	SSD
Worker-3	Physical	2xIntel Xeon E5504	8	24 GB	HDD
Worker-4-6	Physical	Intel Xeon X3430	4	4/8 GB	HDD

## Results and discussion

In this section, we retrieve the monitoring metrics and RTTs of multiple applications running on the same host and perform a variability analysis. Using our methodology, we identify the most relevant monitoring metrics that contribute to performance variability and gain insight into its underlying causes. We then examine the generalizability of our findings across different application instances and worker nodes. Finally, we demonstrate the efficiency of our methodology in various configurations.

### Co-location effect

We study how variability differs when applications operate on isolated resources compared to shared resources. Table 4 displays the changes in variability and the minimum sample sizes required by CONFIRM ($\alpha$ = 95%, *r* = 3%) for applications running on isolated or shared resources in Worker-1. In the case of shared resources, some applications have multiple instances, resulting in multiple values for variability and minimum sample sizes. Our findings reveal a significant increase in CoV and the minimum sample size when resources are shared. This increase in RTT variability occurs due to resource contention when multiple applications are hosted on the same machine. The degree of variability varies among applications, due to their distinct algorithms and resource demands. For example, the performance of the *Upload* application is influenced by both network and computational resources, while the other applications are primarily affected by computational factors. Additionally, *FFT Mock* maintains relatively low variability even under shared resource conditions. Therefore, different applications exhibit varying levels of resistance to interference caused by noisy neighbors. The minimum number of samples estimated by CONFIRM underscores the need to collect a larger volume of data during real-time operation when applications share resources. This is essential to capture a broader range of performance fluctuations and ensure the dataset accurately represents the underlying performance dynamics.

**Table 4:** Variability comparison and samples isolated and shared execution at Worker-1

Application	Isolated		Shared	
	CoV	Samples	CoV	Samples
Upload	13.65%	165	33.59%	1162
			32.21%	809
gCTF	19.18%	79	71.07%	1561
			71.14%	1475
Motioncor2	11.93%	41	36.85%	1719
ctffind4	4.47%	15	62.5%	382
FFT mock	2.38%	15	4.84%	20
			5.07%	23

### Monitoring metrics importance

We identify the most strongly correlated monitoring metrics for applications when they are co-located by applying the proposed methodology to each application individually. In *stage 1* (refer to 3.1), we analyze the state of 422 monitoring metrics (for Worker-1) provided by Prometheus when tasks are executed. Approximately 298 of these metrics remain stable over time and show minimal correlation with performance variations, leaving approximately 124 metrics (depending on the application and server) that could potentially be valuable. In *stage 2* (see 3.2), we extract features from each metric, generating 785 features per metric. The subsequent *stage 3* (refer to 3.3) involves correlating these extracted features with RTT and eliminating redundancy, using a threshold of 90% for inter-correlation. This step reduces the number of features per metric from 785 to approximately 15 and further refines it to a single feature by selecting the most strongly correlated one. The features of each monitoring metric are then concentrated in a single dataset to identify the most influential metrics.

The total number of monitoring metrics with nonzero correlations is 90, an 80% reduction from the initial set of metrics. This reduction enables a lighter monitoring system, as agents need fewer metrics to be collected and transmitted to a central server. In addition, the smaller set of metrics accelerates further processing, such as the development of predictive models and scheduling, because there are fewer metrics to process.

**Fig. 3:**
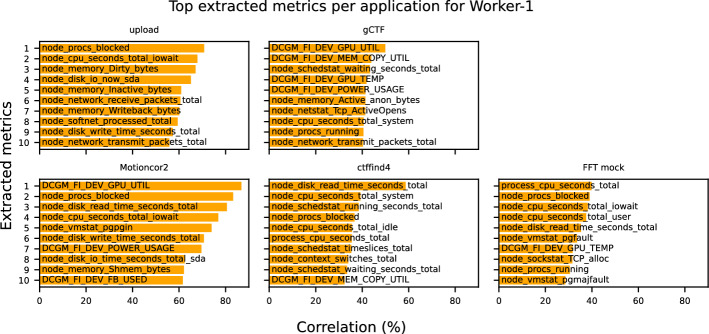
Correlation of the top extracted metrics for each application when co-located on Worker-1

Figure 3 displays the top 10 most correlated metrics for each application hosted on Worker-1. Even through the applications *upload*, *gCTF* and *FFT Mock* have multiple instances, only the most correlated monitoring metrics of the first instance are displayed. We find that different monitoring metrics are most strongly correlated with performance variability for each application, indicating that profiling each application demands a distinct set of metrics. The correlation of the monitoring metrics ranges from 29% to 87%. The metrics extracted during the execution of tasks provide us with the following insights into the behavior of each application:

- ***Upload***: The presence of monitoring metrics node_procs_blocked and node_cpu_seconds_total_iowait suggests that CPU resource contention and I/O wait times are critical, likely due to blocked processes waiting for disk or network I/O. Memory management also plays a significant role, with node_memory_Dirty_bytes, node_memory_Inactive_bytes, and node_memory_Writeback_bytes, indicating that the application is influenced by how the system handles dirty (modified) memory pages and memory writeback processes. Disk-related metrics, including node_disk_io_now and node_disk_write_time_seconds_total, point to active disk I/O operations affecting performance. Network activity, represented by node_network_receive_packets_total, node_network_transmit_packets_total, and node_softnet_processed_total, emphasizes the importance of data transmission and packet processing. Together, these metrics reveal that the application is heavily dependent on resource utilization across CPU, memory, disk, and network subsystems.
- ***MotionCor2***: GPU-related monitoring metrics (DCGM_FI_DEV_GPU_UTIL, DCGM_FI_DEV_POWER_USAGE, and DCGM_FI_DEV_FB_USED) indicate that the performance of the application is sensitive to GPU utilization, power consumption, and framebuffer memory usage, suggesting it involves GPU-intensive tasks. CPU contention is highlighted by node_procs_blocked and node_cpu_seconds_total_iowait, showing that processes are often blocked or waiting for I/O operations. Disk I/O metrics, including node_disk_read_time_seconds_total, node_disk_write_time_seconds_total, and node_disk_io_time_seconds_total, point to high disk read and write times, which can be a bottleneck. The memory metric node_memory_Shmem_bytes implies a dependency on shared memory usage, and node_vmstat_pgpgin reflects paging activity, which can impact memory efficiency. Together, these metrics suggest that the application is both computation- and I/O-bound, relying heavily on GPU, disk, and memory performance.
- ***FFT Mock***: In this application, the presence of CPU monitoring metrics (process_cpu_seconds_total, node_procs_blocked, node_cpu_seconds_total_iowait, node_cpu_seconds_total_user, and node_procs_running) highlight significant CPU usage, I/O wait times, and process contention, indicating that the application competes for CPU resources with other processes. Disk read latency, captured by node_disk_read_time_seconds_total, points to disk I/O bottlenecks. The paging-related metrics (node_vmstat_pgfault and node_vmstat_pgmajfault), indicate the existence of frequent page faults and major page faults affecting performance. Although the application itself is not GPU-intensive, metrics such as DCGM_FI_DEV_GPU_TEMP suggest it is affected by the resource usage of other GPU-based applications running on the same host. This can happen through the use of other resources (i.e., CPU). Network-related contention is also reflected in node_sockstat_TCP_alloc, which tracks TCP socket allocations, potentially impacted by both CPU and I/O load. Therefore, the application performance is influenced by both CPU and I/O-related factors.
- ***gCTF***: GPU-related metrics (DCGM_FI_DEV_POWER_USAGE, DCGM_FI_DEV_GPU_UTIL, DCGM_FI_DEV_MEM_COPY_UTIL, and DCGM_FI_DEV_GPU_TEMP) reveal that the GPU utilization and memory copy operations play a key role, while temperature increases may impact performance. CPU metrics (CPU time in system mode (node_cpu_seconds_total_system, node_schedstat_waiting_seconds_total and node_procs_running), suggest the application is often waiting for CPU resources, likely due to process scheduling and competition. Memory usage, particularly active anonymous memory (node_memory_Active_anon_bytes), shows memory demand affecting overall performance. Network activity, seen in node_netstat_Tcp_ActiveOpens and node_network_transmit_packets_total_lo, points to active network connections and data transmission, contributing to performance variability. Overall, the application performance is driven by both GPU and CPU usage as well as memory.
- ***ctffind4***: High disk read times (node_disk_read_time_seconds_total_sda) suggest the application frequently accesses the disk, impacting performance. CPU system time (node_cpu_seconds_total_system) indicates intensive kernel-level processing, while the idle CPU time (node_cpu_seconds_total_idle) shows periods of CPU inactivity, possibly waiting for I/O operations. Frequent task scheduling and context switching (node_schedstat_running_seconds_total, node_schedstat_timeslices_total, and node_context_switches_total) point to substantial overhead from process management. Processes being blocked (node_procs_blocked) suggest delays due to resource contention. The process_cpu_seconds_total metric highlights the total CPU usage by the application. Additionally, waiting times for CPU access (node_schedstat_waiting_seconds_total) reveal scheduling delays, contributing to variability in the application performance. Although *ctffind4* is not GPU-based, GPU activity (e.g., from *MotionCor2*) can affect RTT due to subsequent resource usage (e.g., CPU, memory). In general, the application completion time is influenced by significant CPU scheduling and disk I/O activity.

**Fig. 4:**
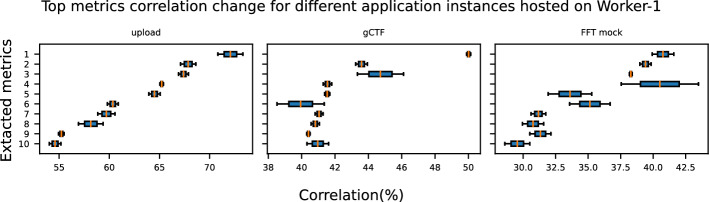
Correlation change between different application instances running on Worker-1. The y-axis of each subplot represents the metric-IDs of the corresponding application instance shown in Fig. 3

We analyze the generalization of the identified monitoring metrics of one instance to the rest instances of the same application running on the same host. Figure 4 shows the correlation differences of the first application instance and the second one for *Upload*, *gCTF*, and *FFT Mock* on Worker-1. This is achieved by skipping step 3d (3.3), which removes redundant metrics, for the second instance of each application. We observe that the correlation differences of the identified monitoring metrics of each application instance are quite low. The maximum correlation differences for each application are 2.49% (*Upload*), 2.83% (*gCTF*), and 5.94% (*FFT Mock*). Therefore, we conclude that instances of the same application running on the same worker node exhibit similar behavior, and the metrics of one instance can be representative of the others.

### Generalization to other servers

**Table 5:** Variability comparison among workers

Application	Worker-1		Worker-2		Worker-3	
	CoV	Mean	CoV	Mean	CoV	Mean
Upload	33.59%	35.77s	21.54%	10.83s	21.80%	30.70s
gCTF	71.07%	4.81s	–	–	–	–
Motioncor2	36.85%	56.62s	–	–	–	–
ctffind4	62.50%	1.75s	10.41%	0.96s	7.10%	3.44s
FFT mock	4.84%	40.38s	6.62%	20.76s	6.68%	58.21s

In this subsection, we analyze the variability when applications are deployed on Worker-2 and Worker-3. It is important to note that *MotionCor2* and *gCTF* are excluded from these workers due to the absence of GPU support. Table 5 presents the CoV and the mean RTT for all applications on each target worker node. The results indicate that *upload* and *ctffind4* exhibit significantly higher variability in Worker-1 compared to other nodes. For *FFT Mock*, the variability remains similar across all worker nodes but reaches its peak on Worker-2. The increased variability on Worker-1 is attributed to CPU multithreading capabilities, which have been shown to increase performance fluctuations [5]. Additionally, we observe distinct differences in mean RTT across the worker nodes. These differences arise from the different hardware (e.g., CPU models) and software configurations (e.g., OS kernel version) of the worker nodes, which influence application execution times. Moreover, the number of instances hosted on each worker node varies, resulting in different co-location scenarios that affect both performance variability and mean RTT.

**Fig. 5:**
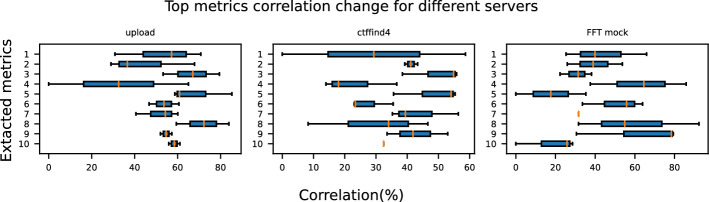
Correlation change of metrics extracted for a particular application from Worker-1 compared to Worker-2 and Worker-3. The y-axis of each subplot represents the metric-IDs of the corresponding application instance shown in Fig. 3

We investigate the generalization of the extracted metrics from Worker-1 to Worker-2 and Worker-3. Figure 5 displays the comparison of the correlations for the top 10 extracted metrics between the applications when hosted in Worker-1, compared to their performance on Worker-2 and Worker-3. We observe significant variations in both the correlation values (indicated by the standard deviation of the error bars) and the ranking of key metrics (represented by the mean of the error bars) between the worker nodes. Specifically, the highest correlation differences are 65%, 58%, and 60% for *upload*, *ctffind4*, and *FFT Mock*, respectively.

The observed differences in metric correlations across servers stem from variations in hardware configurations, software environments, and co-location scenarios:

- **Impact of hardware and software configurations:** Different server configurations, such as CPU models, memory architecture, and kernel versions, influence the variability of performance. Hardware components with different isolation mechanisms (e.g., hyperthreading, cache policies) introduce variability in the degree of resource contention, which, in turn, alters the importance of specific monitoring metrics. For example, servers with enabled CPU multithreading show increased performance variability [5]. Furthermore, servers with smaller resource capacity may deplete resources faster, resulting in more intense performance variability under heavy workloads. In this way, different monitoring metrics gain a higher correlation per case.
- **Effect of co-location scenarios:** Co-location scenarios vary across worker nodes due to differences in the number and type of applications hosted simultaneously. These variations lead to different levels of resource interference that affect the relevance and importance of specific monitoring metrics. For instance, a worker node running multiple I/O-intensive applications will exhibit a stronger correlation with disk-related metrics. In contrast, a node that executes CPU-bound applications will highlight metrics related to processor performance.

Therefore, it is crucial to perform the metric extraction methodology separately for the application deployed on each node to ensure accurate results.

### Number of monitoring metrics

**Fig. 6:**
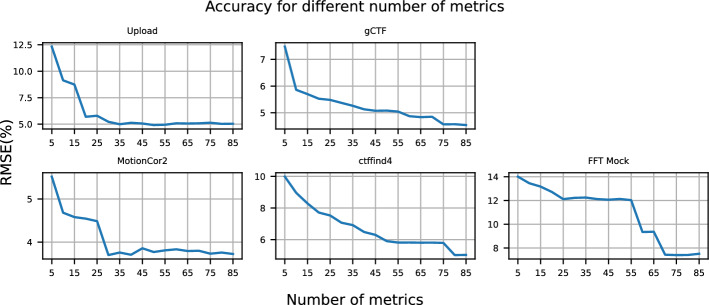
Accuracy of XGBoost for each application on Worker-1 when the used number of monitoring metrics is gradually increased

To determine the number of monitoring metrics required to capture the performance variability of applications, we build an XGBoost model [32] for each application. XGBoost is selected for its ability to capture nonlinear relationships, high computational efficiency, handling of missing data, and built-in regularization to mitigate overfitting. Even though we are not making future predictions (as we use the current state of monitoring metrics to estimate RTT), machine learning models consider both nonlinear interactions between input features and the target variable, and they treat input features collectively rather than independently. This highlights the limitations of our methodology, which treats monitoring metrics linearly and independently. The model uses the monitoring metric values (represented by the temporal and statistical features extracted using tsfresh) as input and predicts RTT. Both metrics and RTT are normalized between 0 and 1 using MinMax normalization, and outliers are removed by excluding samples with a z-score greater than 3. The dataset is divided into a training set (80%) and a test set (20%). The accuracy of the generated models is evaluated using the root-mean-square error (RMSE). Figure 6 illustrates the RMSE of XGBoost for each application as the number of monitoring metrics increases. We begin by training the model with the five most correlated metrics and progressively add the next five most correlated ones, continuing this process until all metrics are included.

We observe that XGBoost achieves a low prediction error for RTT across all applications using the identified monitoring metrics. The model for *FFT Mock* exhibits the highest error, reaching 8% when all metrics are used. This supports the importance of these metrics in capturing key factors that contribute to performance degradation. In addition, a gradual reduction in RMSE is observed in all applications as more metrics are used. The number of metrics required to capture performance variability differs for each application. For example, in the cases of *gCTF*, *ctffind4*, and *FFT Mock*, the error continues to decrease as more metrics are added. In contrast, for *Upload* and *MotionCor2*, no significant improvement in accuracy is observed after 20 and 25 metrics, respectively. This suggests that different sets of monitoring metrics are necessary to generate individual profiles for each application, as each has unique resource demands. For *FFT Mock*, we observe that RMSE reaches a local minimum at 22 metrics and remains stable until it drops further after 48 metrics. This indicates that metrics between 22 and 48 contribute little additional information to the model and should be ranked lower in importance. We attribute this to nonlinear relationships between metrics and RTT, which are not captured by Pearson correlation.

### Prediction power of the extracted monitoring metrics

**Fig. 7:**
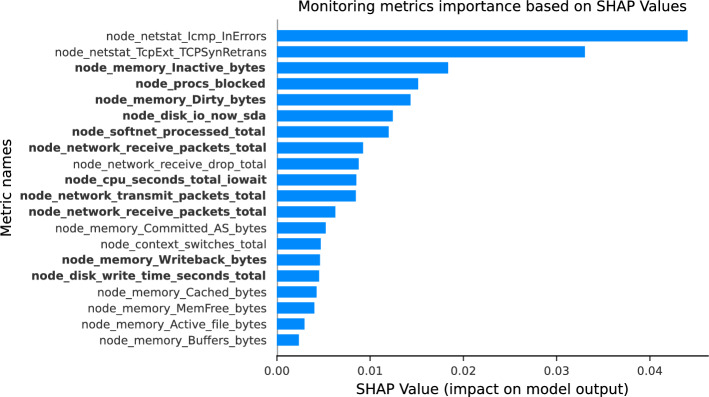
Monitoring metric importance based on SHAP values for an XGBoost model trained on 20 metrics for the Upload application running on Worker-1. The top 10 metrics identified by our methodology are highlighted in bold

We evaluate the actual predictive power of the monitoring metrics using SHAP [33], a method based on cooperative game theory that quantifies the contribution of each feature to the model predictions. SHAP provides insights into both the importance and the individual impact of features within machine learning models. We apply SHAP to the previously trained XGBoost model for the *Upload* application running on Worker-1, using the 20 monitoring metrics identified as optimal in the previous subsection. Figure 7 visualizes the contributions of each monitoring metric to XGBoost predictions through SHAP values, with the top 10 metrics identified by our methodology highlighted in bold.

Despite successfully identifying the 20 most correlated metrics needed to estimate RTT, the correlation rankings do not align with the SHAP value rankings. This discrepancy arises from the following key factors:

1. **Feature interactions:** SHAP values account for both linear and nonlinear interactions between features, which linear correlation alone cannot capture. For instance, a feature with moderate correlation may, in combination with others, have a larger impact on the predictions of the model.
2. **Global vs. local importance:** SHAP measures global feature importance across the entire dataset, reflecting how consistently each metric contributes to predictions. A metric highly correlated with application completion time might not consistently contribute to predictions in all cases.
3. **Model complexity:** XGBoost is a nonlinear model, and SHAP values can uncover nonlinear patterns that may not be evident from a linear correlation analysis. Thus, features with low Pearson correlation can have a significant impact due to nonlinear effects.

Our methodology effectively reduces the initial 442 metrics of Worker-1 from Prometheus to a meaningful subset of 90 metrics by filtering out irrelevant and redundant metrics. This subset is sufficient to explain the behavior of the application. We employ Pearson correlation for this purpose because of its computational efficiency, enabling rapid identification of metrics that are linearly correlated with performance variability. However, it is important to acknowledge the limitations of Pearson correlation. While it is effective at identifying linear relationships, it cannot capture nonlinear interactions or complex dependencies between features, which may also contribute to performance variability.

Consequently, although Pearson correlation provides a computationally efficient way to filter and rank metrics, its simplicity means that the resulting ranking may not fully reflect the true predictive power of the metrics. For instance, advanced techniques like SHAP, which consider feature interactions and non-linear effects, can reveal additional insights into the importance of metrics.

This observation does not invalidate the methodology, but highlights a trade-off between computational efficiency and the depth of analysis. In real-time systems or large-scale deployments, the computational simplicity of Pearson correlation makes it a practical choice for initial filtering. However, in scenarios where computational resources and time allow, the methodology could be extended to include more sophisticated methods for feature evaluation, such as other nonlinear analysis techniques. These methods could refine the subset of metrics by uncovering relationships that Pearson correlation could overlook.

The methodology can be applied to build performance predictors by identifying the most correlated monitoring metrics in the time window prior to task submission (e.g., 5 s before submission). This approach pinpoints the relevant metrics to use as input for machine learning models. Additionally, the tsfresh extracted features from time-series data can be directly employed to generate non-sequential models, such as support vector machines or decision trees. This enables the estimation of the task RTT by retrieving the state of monitoring metrics before submission. Such insights are especially valuable for scheduling decisions, where the optimal worker node needs to be identified for incoming requests.

### Prediction accuracy of multiple tsfresh features

**Fig. 8:**
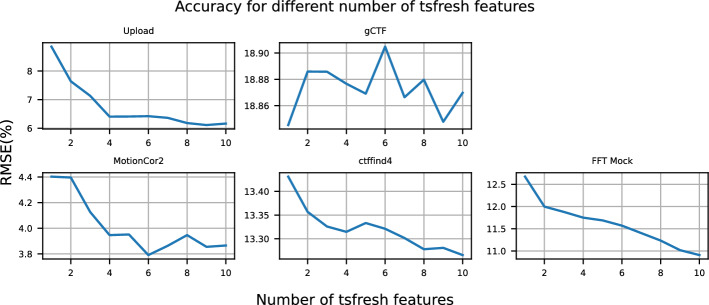
RMSE of XGBoost when additional tsfresh features are included for the top 10 most correlated metrics of each application running on Worker-1

Our methodology initially extracts multiple features from each monitoring metric using the tsfresh library, but ultimately selects only the most correlated feature with RTT. We investigate whether using additional tsfresh features per monitoring metric adds predictive power. To explore this, we follow a similar approach to subsection 5.4, where we develop a XGBoost model for each application using the top 10 most important metrics. We begin by training the models with a single tsfresh feature per monitoring metric and then gradually add the next most correlated one, until ten features per metric are added.

Figure 8 illustrates the RMSE of the XGBoost models for each application as the number of tsfresh features per monitoring metric increases. We observe that for *Upload*, *MotionCor2*, *ctffind4*, and *FFT Mock*, the RMSE gradually decreases as more tsfresh features are included. However, the reduction in RMSE is relatively small. In the case of *gCTF*, adding more features results in slight fluctuations in RMSE, indicating that including additional tsfresh features introduces noise, which can increase prediction error. These results suggest that using a single tsfresh feature per monitoring metric is generally sufficient when transforming the time series into features. Adding more features beyond this point does not significantly improve performance and may, in some cases, degrade it due to added noise.

### Proportion of shared metrics across applications

**Fig. 9:**
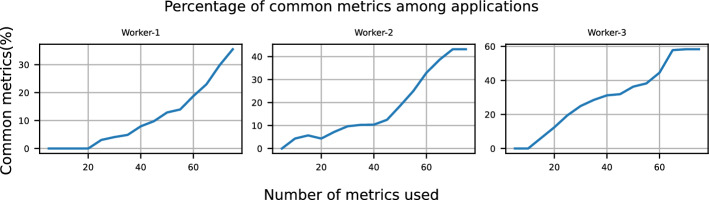
Percentage of common metrics among all applications as the number of monitoring metrics per application increases

We examine how the proportion of shared monitoring metrics between all applications deployed on a worker node evolves as more metrics are considered. To do this, we start by selecting the five most important metrics for each application running on a specific worker node, then calculate the percentage of shared metrics that are important across all applications. We repeat this process by adding the next five most correlated metrics until all metrics are included for all applications. The results for each worker node are shown in Fig. 9.

We observe that initially (i.e., the top 10 metrics), the most important metrics are completely distinct across applications on all worker nodes. As the number of metrics considered increases, the percentage of common metrics increases linearly. This trend is consistent across all worker nodes. When all metrics are included, the maximum proportion of shared metrics reaches 35% for Worker-1, 45% for Worker-2, and 60% for Worker-3. The variation in the maximum proportion of shared metrics across worker nodes is primarily due to differences in co-location scenarios. For example, Worker-1 also hosts *gCTF* and *MotionCor2*, which are not present at the other nodes.

Furthermore, the proportion of common metrics never reaches 100%, even when all metrics are considered. This is due to two key reasons: first, metrics that are correlated with RTT for one application may be entirely irrelevant for another. Secondly, important metrics for one application may be discarded as redundant for another. This occurs during step 3d of our methodology, where only the most correlated metric with RTT is retained among the metrics that are highly correlated with each other. These findings further support the importance of creating individual profiles for each application, as the monitoring metrics of each application remain distinct even when all relevant monitoring metrics are included.

### Occurrence of tsfresh features among monitoring metrics

**Fig. 10:**
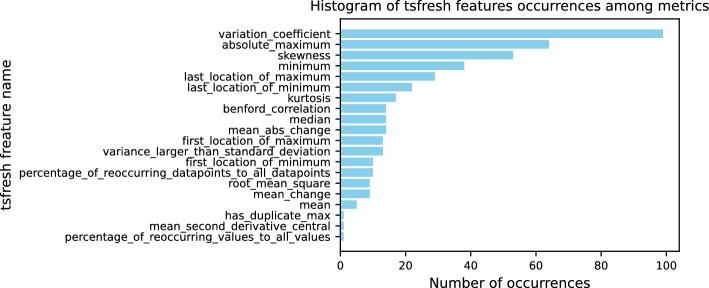
Number of occurrences of tsfresh features which acquire the highest correlation on all monitoring metrics of Worker-1

Tsfresh extracts approximately 785 temporal and statistical features, depending on the library configuration used. Among these features, some are automatically discarded by tsfresh as irrelevant to the given time series (e.g., CPU usage) and the target variable (e.g., RTT). Additionally, since we correlate the state of the monitoring metrics during task execution with their RTT, we remove length-based features (as explained in stage 2 of our methodology). We count the occurrences of the tsfresh features that appear as the most correlated across all monitoring metrics, representing the features that carry the most information. Figure 10 illustrates the frequency of occurrence of each selected tsfresh feature in all monitoring metrics. For each monitoring metric, the calculated tsfresh feature with the highest correlation value is considered the selected one and is included in the histogram. We observe that 22 tsfresh features remain relevant across monitoring metrics. Although common features such as *minimum*, *absolute maximum*, *median*, and *mean* are commonly used to transform time series into the feature domain for monitoring, other less conventional features also gain significant importance for specific metrics. This emphasizes the importance of extracting a broad range of statistical and temporal features to capture the most meaningful insights from time-series data.

It is important to note that the coefficient of variance is the most correlated feature of the monitoring metrics with the application RTT. This means that the relative variability of the monitoring metrics is a strong indicator of performance. The coefficient of variance indicates the degree of fluctuation of a monitoring metric relative to its mean. A high coefficient of variance in the monitoring metrics might indicate periods of instability, bottlenecks, or inconsistent resource availability, all of which contribute to longer completion times. A low coefficient of variance would imply more consistent performance, where resource usage is stable, often resulting in faster completion times.

### Lessons learned

From these results, we derive the following guidelines to generalize the proposed methodology to diverse contexts and conditions:

1. **Application profiling:** Monitoring metrics are closely correlated with the performance variability of running applications. Each application exhibits strong correlations with a unique subset of monitoring metrics, which may vary in both type and quantity. These metrics provide valuable insight into the behavior of target applications and have the potential to be used to forecast application completion times.
2. **Node-specific metric extraction:** The heterogeneity of worker nodes, in terms of hardware (e.g., CPU architecture) and software (e.g., operating systems), plays a critical role in shaping the relevance and behavior of performance metrics. To account for these variations, the metric extraction must be performed independently for each node, ensuring accuracy in the analysis.
3. **Adaptation to co-location scenarios:** Co-location of multiple application instances has a significant impact on performance variability due to resource contention. It is crucial to continuously update the correlation between monitoring metrics and application performance to capture the dynamic resource sharing conditions specific to each node.
4. **Correlation algorithms:** Pearson correlation is computationally efficient for filtering and ranking metrics, making it an effective tool for quickly removing irrelevant metrics and accelerating post-processing. However, its simplicity limits its ability to capture nonlinear relationships and feature interactions. Techniques such as Spearman correlation, which account for these complexities, can provide deeper insight into the metric importance and improve the accuracy of the analysis.
5. **Time-series features extraction:** To transform monitoring metrics from the time domain to the feature domain, it is essential to extract the most representative features (e.g., coefficient of variation, maximum, mean). Our findings indicate that retaining a single well-selected feature per metric is sufficient to represent its behavior effectively.

Following these principles, the proposed methodology can be adapted to a variety of server configurations and application scenarios, providing a flexible and robust framework for the analysis of performance variability.

## Conclusions

In this paper, we investigated the performance variability in edge computing system using single-particle analysis (SPA) applications, where collocated tasks contend for compute and network resources. This results in significant fluctuations in task execution performance. We developed a methodology that correlates monitoring metrics with round-trip time (RTT) in edge computing tasks by extracting time-series features. This method identifies the most critical metrics that influence performance variability during task execution. Our results demonstrate that individual application performance is strongly tied to specific metrics, highlighting the need for customized profiling for each application. These insights provide a deeper understanding of the underlying causes of performance degradation and show that different applications require varying numbers of metrics to accurately capture performance variability. By generating simple machine learning models for each application, we further demonstrated that the identified metrics have predictive power, enabling the estimation of RTT and offering potential for performance forecasting.

For future work, we aim to incorporate advanced correlation algorithms to better capture both linear and nonlinear relationships between monitoring metrics and RTT variability. Additionally, we plan to use historical monitoring data to develop predictive models that can be integrated into the Kubernetes scheduler. This integration has the potential to facilitate more informed scheduling decisions and improve overall application performance.

## Data Availability

The code and datasets generated and/or analyzed during the current study are available in the Zenodo [https://doi.org/10.5281/zenodo.15359602].

## Abbreviations

OPEX: Operational expenses
RTT: Round-trip time
SLA: Service-level agreement
SPA: Single-particle analysis
EM: Electron microscopy
HPC: High-performance computing
OS: Operating system
TSN: Time-sensitive networking
CoV: Coefficient of variation
GPU: Graphics processing unit
CPU: Central processing unit
API: Application programming interface
SHAP: SHapley Additive exPlanations
RMSE: Root-mean-square error
FFT: Fast Fourier transform
